# Does Whole Grain Consumption Alter Gut Microbiota and Satiety?

**DOI:** 10.3390/healthcare3020364

**Published:** 2015-05-29

**Authors:** Danielle N. Cooper, Roy J. Martin, Nancy L. Keim

**Affiliations:** 1 Department of Nutrition, University of California at Davis, 1 Shields Ave, Davis, CA 95616, USA; E-Mails: dncooper@ucdavis.edu (D.N.C.); roy.martin@ars.usda.gov (R.J.M.); 2 USDA-ARS, Western Human Nutrition Research Center, 430 West Health Sciences Drive, Davis, CA 95616, USA

**Keywords:** gut microbiota, satiety, whole grains, VAS appetite assessment, short chain fatty acids, bile acids, obesity

## Abstract

This review summarizes recent studies examining whole grain consumption and its effect on gut microbiota and satiety in healthy humans. Studies comparing whole grains to their refined grain counterparts were considered, as were studies comparing different grain types. Possible mechanisms linking microbial metabolism and satiety are described. Clinical trials show that whole grain wheat, maize, and barley alter the human gut microbiota, but these findings are based on a few studies that do not include satiety components, so no functional claims between microbiota and satiety can be made. Ten satiety trials were evaluated and provide evidence that whole oats, barley, and rye can increase satiety, whereas the evidence for whole wheat and maize is not compelling. There are many gaps in the literature; no one clinical trial has examined the effects of whole grains on satiety and gut microbiota together. Once understanding the impact of whole grains on satiety and microbiota is more developed, then particular grains might be used for better appetite control. With this information at hand, healthcare professionals could make individual dietary recommendations that promote satiety and contribute to weight control.

## 1. Introduction

The aim of this review is to evaluate the evidence for whole grain effects on satiety and to discuss if such effects might be attributed to the gut microbiota. We will define whole grains and describe consumption patterns in the United States adult population. To understand the relationship between microbiota and satiety, it is important to lay a foundation on the characterization of the gut microbiota and to provide a theoretical framework relevant to understanding potential mechanisms, whereby the microbiota may affect satiety. Finally, recent studies are presented that address the effect of consuming whole grains on gut microbiota or satiety. Whole grains comprise a diverse group of staple foods, and in this review, the more commonly-consumed grains—wheat, corn, rice, oats, barley and rye—are included. We conclude by discussing gaps in the literature and suggesting areas for future research.

## 2. Background: Whole Grains

In the United States, the Dietary Guidelines for Americans, which is jointly published by the United States Department of Agriculture (USDA) and the U.S. Department of Health and Human Services (HHS) recommend that half (3–5 servings or 48–80 g) of one’s daily grain intake should be in the form of whole grains. Internationally, whole grain intake is encouraged, although the amounts vary among different countries [1]. These recommendations are based on a body of literature that suggests that there are health benefits associated with the consumption of whole grains and epidemiological studies that show correlations between whole grain consumption and better health [2]. Many excellent reviews have detailed the effects of whole grain consumption on chronic diseases, linking the functional components of whole grains to their beneficial effects on cardiovascular diseases, metabolic syndrome, type 2 diabetes and some forms of cancer [3,4,5,6,7,8,9,10,11]. There is also interest in determining if whole grain contributes to body weight management: weight loss, a decrease in fat mass or prevention of excess weight gain. Epidemiological studies show that consumption of whole grains is correlated with leanness in adults; however, randomized controlled trials have demonstrated mixed results [12,13,14]. A recent meta-analysis reviewing 26 studies reported no difference in body weight, but did report a modest decrease in body fat with whole grain consumption compared to the control. In this analysis, most studies averaged 4–6 weeks in duration, which may not be long enough to see true changes in body weight [12]. The composition of whole grains in various study diets may also complicate interpretation, since each type of whole grain is unique and offers different levels, types or ratios of fiber, micronutrients, macronutrients and bioactives. Differences in energy intake, influenced by levels of satiation, may result from differences in whole grains. Further, differences in satiation may be mediated by the gut microbiota [15], which was not evaluated in the meta-analysis. Any or all of these factors might account for some of the conflicting results reported regarding body weight and composition in response to consumption of whole grains [6,8,16,17].

## 3. Defining Whole Grains

The American Association of Cereal Chemists International states that whole grains are “intact, ground, cracked or flaked fruit of the grain whose principal components, the starchy endosperm, germ and bran, are present in the same relative proportions as they exist in the intact grain” [18]. The Europe-based HEALTHGRAIN consortium defines whole grains for food consumption as “consisting of the intact, ground, cracked or flaked kernel after the removal of inedible parts such as the hull and husk. The principle anatomical components—the endosperm, germ and bran—are to be present in the same relative proportions as they exist in the intact kernel. Small losses of components, *i.e.*, <2% of the germ or <10% of the bran, which may occur through processing methods consistent with safety and quality, are allowed” [19]. These definitions coincide for the most part, and there is little disagreement in the scientific community about what is considered a whole grain. Wheat (Triticum spp.), corn/maize (Zea mays), rice (Oryza spp.), oats (Avena spp.), barley (Hordeum spp.) and rye (Secale cereal) are the most commonly studied and consumed grains [5].

## 4. Whole Grain Consumption in the United States

**Figure 1:**
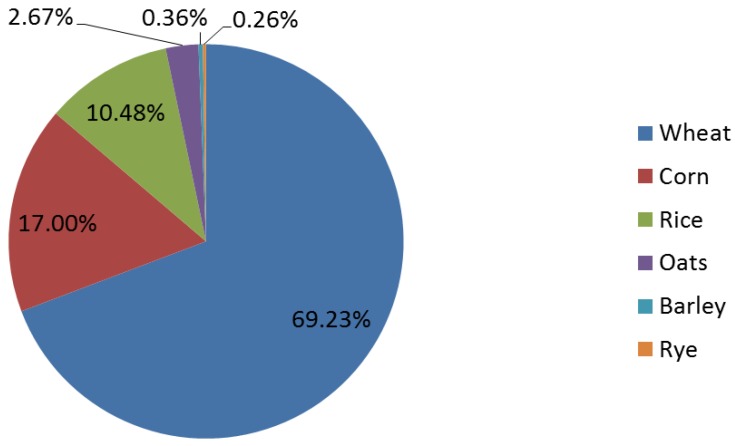
Percent of per capita availability of cereal grains in the United States. Data from the USDA Economic Research Service, 2012 [20].

The grains discussed in this review are identified by the United States Department of Agriculture (USDA) Economic Research Service to have the highest annual per capita availability (Figure 1). In the U.S., wheat (including white, whole and durum wheat) had the greatest availability at 61 kilograms per person.

These data report grain availability in the U.S. food supply, and they do not reflect what is actually consumed or differentiate between whole and refined grains. According to the National Health and Nutrition Examination Survey (NHANES) conducted in the U.S. during 2009–2010, adults 19–50 years of age only consume an average of 0.61 ± 0.02 ounce equivalents per day of whole grains, much less than the minimum recommended amount of three ounce equivalents per day. One ounce equivalent is approximately equal to 16 grams of whole grain, so the deficit is about 38 grams of whole grain per day. There are no data available breaking down exactly what percent each grain contributed to the average serving [21].

**Figure 2:**
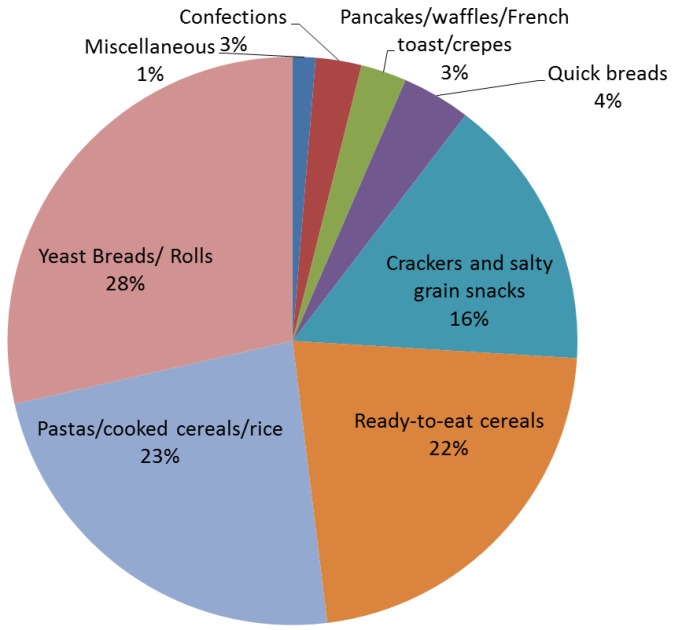
Products that contributed to adult whole grain intake. Data represent the average consumption of different categories of whole grain foods from the National Health and Nutrition Examination Survey (NHANES) 2009–2010 [11].

Whole grains were primarily consumed as yeast breads/rolls, pastas/cooked cereals/rice and as ready-to-eat breakfast cereals (Figure 2).

## 5. A Primer on Gastrointestinal Microbiota

Within the human gastrointestinal (GI) system, there are as many as 100 trillion bacterial cells that make up the human gut microbiome [22]. Many of these organisms subsist by using non-digestible carbohydrates as their carbon source and fermenting them to create energy. Whereas humans do not possess the enzymes needed to breakdown compounds, such as cellulose, inulin, xylans and resistant starch, many gut bacteria can via fermentation [23]. These compounds that are not digestible by the host can promote bacterial growth, and if bacterial metabolism of the compound confers a health benefit to the host, they can be referred to as prebiotics [24]. Products of bacterial fermentation include molecules, such as short chain fatty acids (SCFA), which include acetate, propionate and butyrate, as well as others [25]. SCFA are not only an energy source for bacteria, but can also be utilized by the human host. For example, the colonic epithelium can utilize butyrate, whereas acetate and propionate are more frequently used in peripheral tissues [23].

While every human has gut microbiota, the composition, concentration and diversity of the bacteria differ between individuals [26]. Due to this variation between individuals, many studies that utilize microbial characterization use crossover study designs to reduce the chance of confounding covariates and to preserve statistical power, so that fewer subjects are needed [27,28,29]. When the microbial population is assessed, baseline and post intervention changes, or shifts, in microbiota can be detected on an individual or treatment group basis [30]. Shifts in microbiota do not necessarily benefit or detract from the host; shifts merely denote that there was a change, and further analysis is necessary to evaluate the effect of the change [27,28]. There are a number of methods used to characterize fecal microbiota, each with its own strengths and weaknesses [29]. Some of the more common approaches to characterization of microbiota include fluorescence *in situ* hybridization (FISH), high throughput sequencing by synthesis, or pyrosequencing, of the bacterial 16s ribosomal RNA gene, quantitative polymerase chain reaction (qPCR) and culturing bacteria [29,31,32]. Further evaluation can be done by looking at products of microbial metabolism in blood, urine and stool using methods, such as high-performance liquid chromatography and gas chromatography-mass spectrometry [33,34].

Recently, there has been a great deal of interest in delineating the effects that gut microbiota have on the parameters of health. Two phyla of bacteria that are often of interest are the largely Gram-positive endospore producing Firmicutes and the Gram-negative and anaerobic Bacteroidetes [35,36]. Plant-based diets higher in fiber seem to shift the ratio toward lower Firmicutes and greater Bacteroidetes [37]. The benefits of this shift are under debate, but it is proposed that Firmicutes are associated with impaired gut barrier function, serum LPS levels and inflammatory responses that may lead to metabolic dysfunction [38]. However, the ratio of Firmicutes to Bacteroidetes may vary with a number of non-diet-related factors; one study showed that the ratio of Firmicutes to Bacteroidetes changes with age [39,40]. Increases in overall microbial diversity may be beneficial, as some evidence suggests that lean individuals have a more diverse microbiota compared to obese individuals [41].

Researchers have yet to definitively show how increased diversity or shifts in the ratios of specific groups of gut microbes relate to colonic fermentation and its biomarkers; similarly, a definitive understanding of how these differences may impact health is not yet available [36,40,42]. One potential explanation as to why changes in gut microbiota are so challenging to connect to fermentation or health outcomes is that many studies have focused on characterizing bacteria at the level of phyla, class, order, family or genera rather than at the species level. Studies are finding that bacteria of the same genus may have different effects on the host [43]. Adding further complexity, many bacteria can utilize different substrates depending on environmental factors and the availability of substrates, such as prebiotics, so it may not always be possible to empirically determine the metabolic contribution of a particular bacterial species [35,44]. Some research is being done using metabolomics and metaproteomics to functionally characterize the proteins synthesized by the gut microbiota in an effort to understand and quantify the actual metabolic activity of a subject’s bacterial community [45,46]. Function-centered approaches may prove to be the future of gut microbial characterization. As information accrues and more insightful approaches are found, the closer we come to correlating changes in the appearance, metabolism or concentration of bacteria and health outcomes.

## 6. Link between Whole Grains, Gastrointestinal Microbiota and Satiety

Due to a growing body of literature on the importance and function of human gut microbiota, scientists are attempting to describe mechanisms that may relate shifts in microbiota to health outcomes. One reason it is believed that there may be a link between shifts in microbiota and satiety is that the satiating effect seen with whole grains can often not be explained by fiber content alone [47].While it is possible that this effect is mediated by another property of whole grains, such as bioactives, there is growing interest in, and evidence of, a potential link between satiety and microbiota [48,49]. To date, very few bacteria have been identified that have a consistent and repeatable impact on specific parameters of health [29,50,51]. A reason for this may be that each species of bacteria residing in the human gut is a member of a community and so functions according to the dictates and strictures of that community. Therefore, it is possible that the same species of bacteria may have different metabolic activity and function depending on its community [52]. This means that a shift in microbial species may not be required to see an effect on satiety; a shift in gene expression may be all that is necessary. In any given person, there are around 540,000 microbial genes that represent the dominate microbes of that ecosystem; only approximately 55% of these genes are shared by at least 50% of the human population [53]. Given the diversity of bacterial species and genes and the lack of certainty regarding how the microbiota could impact satiety, further research is needed to arrive at definitive answers on how a change in gene expression or a shift in a species, genus or phyla of bacteria could affect all, or even most, individuals. Presented below are some potential mechanisms describing how whole grains may influence satiety.

### 6.1. Bioactive Components of Whole Grains

Whole grains contain a plethora of bioactive compounds that, potentially, could affect energy metabolism, weight regulation and food intake [54]. In addition to dietary fibers, discussed in detail below, polyphenol compounds have been shown to have potential to affect neuropeptides involved in food intake and satiety. Evidence from animal studies suggests that polyphenols may act on insulin-signaling pathways to modulate insulin signaling in the brain, and they may exert inhibitory effects on gene expression of orexigenic neuropeptides [55]. However, it must be emphasized that the data currently available are based on studies using food matrices other than grains.

### 6.2. Short Chain Fatty Acids from the Breakdown of Dietary Fibers

Because whole grains contain fibers that are indigestible to humans (such as arabinoxylans or β-glucan), these fibers make it to the distal small or large intestine more or less intact and can be fermented by the resident bacteria [56]. Fermentation can yield products that are recognized and utilized by the human host [57]. These products include short chain fatty acids (SCFA), most commonly acetate, propionate and butyrate. A large portion of the locally-produced butyrate is utilized by the colon, whereas propionate is used by the liver and peripheral tissues; acetate can cross the blood brain barrier and is utilized as an energy source for glial cells [58,59]. Overall, SCFAs are thought to fulfill about 10% of total body energy needs [58]. Given the importance and contribution of SCFAs, one would expect to see more data published delineating how both fecal and circulating SCFAs change relative to dietary interventions. With methodological advancements that increase the sensitivity of detection and accuracy of measuring concentrations of low concentrations of SCFA in the circulation, our understanding of the influence of SCFA on health outcomes will increase.

#### 6.2.1. Butyrate

Butyrate, primarily produced by Firmicutes, such as *Roseburia*, *Eubacterium rectale*, *Faecalibacterium prausnitzii*, as well as many *Clostridium* species, is thought to be a principle fuel source for the cells of the colon [49]. Butyrate has also been shown to help maintain the integrity of the gut epithelial layer, because it can increase the transcription, and, thereby, the production, of the proteins integral to the formation of tight junctions between the colonocytes. With stronger tight junctions, bacterial infiltration at the tight junctions is reduced. Butyrate has been known to reduce gastrointestinal permeability by increasing the activation of peroxisomal proliferator-activated receptor gamma (PPARγ), which is found in colonic epithelial cells and, when activated, reduces or blunts inflammation of the colonocytes. There is also some evidence to suggest that the bacteria that produce butyrate may also upregulate the production of glucagon-like peptide (GLP)-2, which can increase the proliferation of crypt cells [24]. Crypt cells are critical to maintaining a physical barrier at the intestinal lumen [60]. All of these factors together can be thought to help decrease inflammation. A decrease in inflammation, even though it may be sub-clinical, has been shown to improve insulin resistance, which has been linked to increased satiety [24,61,62]. However, until more studies are done to demonstrate that these anti-inflammatory properties of butyrate-producing bacteria are, in fact, influencing satiety, this mechanism is simply theoretical.

#### 6.2.2. Propionate

Propionate is largely produced by bacteria in the Bacteroidetes phyla fermenting carbohydrates and has a documented effect on satiety [49]. Studies have shown that in rodent and human models, propionate has a high affinity for G protein-coupled free fatty acid receptor (FFAR) 2, also known as G protein-coupled receptor (GPCR) 43, on enteroendocrine L cells in the colon. When stimulated, these L cells mediate GLP-1 release. Activation of FFAR3, also known as GPR41, can stimulate the release of peptide YY (PYY), and some evidence suggests that this pathway can be used to stimulate GLP-1 release, as well [58,63]. Activation of FFAR2 on adipocytes can stimulate the release of leptin. Increased levels of GLP-1, PYY and leptin are considered signals of satiation, which have been shown to reduce food intake in both rodents and humans [64,65,66]. GLP-1 acts to decrease stomach acid production, delay gastric emptying and decrease intestinal motility; PYY inhibits gastric motility and emptying and increases water and electrolyte re-absorption in the colon, causing food to stay in the intestine longer; and leptin acts on the arcuate nucleus of the hypothalamus to regulate appetite [58,64,67]. Colonic delivery of propionate reduced energy intake and weight gain in overweight adults [66].

#### 6.2.3. Acetate

Acetate is known to be produced by *Bifidobacterium* and *Ruminococcus bromii* via fermentation of carbohydrates [68]. Total SCFAs have been shown to decrease acute energy intake without increasing the concentration of GLP-1 or PYY in both humans and rodent models [58,59]. Some evidence suggests that this effect could be mediated by increased levels of acetate from colonic fermentation of carbohydrates. In a murine model, it was found that acetate produced in the colon was able to enter the blood stream, cross the blood brain barrier and be taken up in the brain, particularly the hypothalamus. Under the influence of the increased levels of acetate, the mice consumed significantly less food than control mice at both one and two hours post acetate exposure without significant changes in GLP-1 or PYY levels. The investigators hypothesized that this was due to the higher hypothalamic levels of acetate, increasing the oxidative lactate production from the pyruvate recycling pathway, as well as increasing hypothalamic GABAergic neurotransmission. Adenosine triphosphate (ATP) production would be increased by increased oxidative metabolism, thereby decreasing the ratio of adenosine monophosphate (AMP) to ATP. This will cause a decrease in the activity of AMP kinase, which would reduce the inhibition of acetyl-CoA carboxylase, thereby increasing malonyl-CoA concentration and stimulating pro-opiomelanocortin (POMC) neurons, as well as further increasing GABAergic neurotransmission. The increase in GABAergic neurotransmission and POMC neuron activation will instigate a decrease in acute appetite, demonstrating that a process set in motion by colonic acetate can increase satiety without utilizing GLP-1 or PYY [59]. Acetate has been shown to reduce food intake through a central nervous system mechanism in an animal model of obesity [59].

### 6.3. Bile Acids

There are other theories for how shifts in gut microbiota might mediate satiation, but there is considerably less information or studies in support of these theories. One mechanism that may be of interest is the microbial modification of bile acids affecting liver metabolism through dedicated bile acid receptors, such as the farnesoid X receptor and TGR5. Signals from bile acid receptors can affect the rate of gluconeogenesis and glycolysis, thereby changing blood glucose regulation and modifying satiety [49,69,70]. However, there is very little evidence linking whole grain consumption to alterations in the enterohepatic circulation of bile acids and the resulting bile acid profiles. One study showed a decrease in fecal free bile acid concentration in the feces of subjects who consumed a whole grain rye diet compared to a wheat-based diet [71,72]. To confirm a role of bile acids in promoting satiety, more studies would need to be done to evaluate the effect of whole grain consumption on the dynamics of the enterohepatic circulation of bile acids.

## 7. Whole Grain Consumption and Changes in Gastrointestinal Microbiota

Several human diet intervention studies have been conducted to observe changes in the microbial population in response to consumption of either different whole grains or refined *versus* whole grains. These studies show that whole wheat, corn and barley consumption were respectively linked to significant changes in the human gut microbiota. Specific details and results of these studies are described in the text below and in Table 1.

### 7.1. Microbiota Studies: Wheat

Costabile and colleagues [73] examined the effects of whole grain wheat and its ability to modulate human intestinal microbiota. In this study, 100% whole wheat breakfast cereal was compared to a wheat whole bran-based breakfast cereal (Table 1). In response to the whole grain wheat consumption, more *Bifidobacterium* and *Lactobacillus* were present in fecal samples compared to the wheat bran group. This finding is of particular interest, since it suggests that something other than fiber may be causing enrichment of *Bifidobacterium* and *Lactobacillus* in the whole grain intervention [73].

In another wheat intervention, Christensen and co-workers [74] also saw an increase in *Bifidobacterium* on a whole wheat diet. There was a parallel refined wheat intervention, and those subjects had a decrease in the relative abundance of *Bacteroides* compared to baseline. Unlike other studies that have examined microbiota in response to a specific intervention, this study did not use a crossover design, but the parallel treatment intervention does eliminate the risk of carryover effects from the previous intervention.

**Table 1:** Whole grain intervention trials examining microbiota.

Grain	Citation	Study Design	Subjects	Method	Results
**Wheat**	Costabile *et al.*, 2008In text citation: [73]	Randomized crossover design 2 arms: Whole wheat breakfast cereal Whole bran breakfast cereal Duration: 3 weeks per arm	16 females, 15 males Age (years) 20–42 Mean: 25 BMI (kg/m^2^) 20–30	FISH targeting: *Atopobium* group, *Bifidobacterium* spp., *Eubacterium rectale* group, *Clostridium histolyticum* group and *Lactobacillus/Enterococcus*	- Whole grain wheat cereal increased: *Bifidobacterium Lactobacillus* - No change in fecal SCFA, blood glucose, insulin
**Wheat**	Christensen *et al.*, 2013 In text citation: [74]	Open label parallel intervention - Energy-redistricted whole wheat bread, pasta and biscuits providing 105 g whole wheat/day - Energy-redistricted refined wheat Duration: 12 weeks	72 post-menopausal females Age (years) 45–70 BMI (kg/m^2^) 27–37	Quantitative PCR targeting: Bacteroidetes, Firmicutes, *Bacteroides* spp*.*, *Prevotella* spp*.*, *Lactobacillus* spp*.*, Enterobacteriaceae, *Bifidobacterium* spp*.*: - *B. bifidum* - *B. adolescentis* - *B. catenulatum group* - *B. longum*	- Compared to baseline, the whole wheat intervention saw an increase in the relative abundance of *Bifidobacterium* - Compared to baseline, the refined grain wheat intervention saw a decrease in *Bacteroides* - Fecal water increased trans-epithelial resistance, independent of dietary interventions, across a Caco-2 monolayer
**Corn**	Carvalho-Wells *et al.*, 2010 In text citation: [75]	Randomized crossover design 2 arms: Whole grain maize semolina (29.6% whole grain) Refined maize 48 g/d for 3 weeks per arm	21 females, 11 males Age (years) 32 ± 8 BMI (kg/m^2^) 23.3 ± 0.6	FISH targeting: *Bacteroides* spp., *Bifidobacterium* spp., *Clostridium histolyticum/perfringens* subgroup, *Lactobacillus-Enterococcus* subgroup and total bacteria.	- Whole grain maize semolina increased: *Bifidobacterium* - No difference between whole grain and refined grains treatments in fecal SCFA, blood lipids and glucose concentrations and anthropometric measures
**Barley and Rice**	Martinez *et al*., 2013 In text citation: [76]	Randomized crossover design 3 arms: Brown rice Whole grain barley 50/50 brown riceand barley 60 g/d for 4 weeks per arm	17 females, 11 males Age (years) 25.9 ± 5.5 BMI (kg/m^2^) 25.1 ± 4.5	Pyrosequencing	- Whole grain barley increased: Firmicutes, particularly *Blautia* *-* All treatments had a tendency to increase: Firmicutes, particularly *Blautia*; and decrease: Bacteroidetes, particularly *Bacteroides* - No treatment differences in SCFA
**Mixed Whole Grains**	Ampatzolou *et al.*, 2015 In text citation: [77]	Randomized crossover design 2 arms: Diet enriched with whole grains, >80 g/d Diet restricted in whole grains, <16 g/d Diets maintained for 6 weeks/arm	21 females, 12 males Age (years) 40–65 Mean:48.8 BMI (kg/m^2^) 20–35	FISH targeting: *Clostridium coccoides/Eubacterium rectale* group, *Bifidobacterium* genus, *Lactobacillus-Enterococcus* group, *Lactobacillus-Enterococcus* group, *Bacteroides-Prevotella* group, *Clostridium histolyticum* group and *Escherichia coli*	- No differences reported in microbiota - No differences observed in fecal SCFA, blood glucose or lipid concentrations
**Mixed Whole Grains**	Ross *et al.*, 2011 In text citation: [78]	Randomized crossover design 2 arms: Controlled whole grains diet 150 g/d (64% whole grain wheat, 14% barley and rye 13% WG oats, 9% brown rice Controlled refined grain diet (66% refined wheat, 27% white rice, 8% refined maize); diets maintained for 2 weeks/arm	11 females, 6 males Age (years) 34.1 ± 3 BMI (kg/m^2^) 23.1 ± 0.8	Quantitative PCR targeting: total bacteria, Bacteroides, *Bifidobacterium*, *Clostridium coccoides*, *Clostridium leptum*, Enterobacteria, *Enterococcus* spp. and *Lactobacillus*	- Whole grain diet increased *Clostridium leptum,* trend towards an increase in *Enterococcus* spp. (*p* = 0.06) compared to refined grain

### 7.2. Microbiota Studies: Corn

Carvalho-Wells and co-workers [75] evaluated the prebiotic activity of a maize semolina-derived whole grain cereal compared to a non-whole grain maize-derived cereal. An interesting finding of this study was that a low level of whole grain exposure, only 29.6% of the recommended 48-g serving per day, resulted in a significant alteration of the microbiota. After the whole grain maize intervention, subjects had higher mean group levels of fecal *Bifidobacterium* compared to the refined maize intervention. Furthermore, following the whole grain maize intervention and the ensuing three-week wash out, the *Bifidobacterium* levels dropped back to their pre-treatment values. Future research should evaluate the effects of whole grain corn on specific species of *Bifidobacterium*. It would also be interesting to determine if there is a dose response with increased consumption of the whole grain maize.

### 7.3. Microbiota Studies: Barley and Rice

Martinez and co-workers [76] conducted a novel study looking at the effects of brown rice, whole grain barley and a 50/50 combination of brown rice and whole grain barley on fecal microbiota. In response to all three grain treatments, there was a significant increase in bacterial diversity compared to the baseline; however, the grain treatments did not affect species richness. With all grains, there was a tendency for an increase in Firmicutes, particularly in the genus *Blautia*, but the increase was only significant with the barley treatment. Both barley and the barley-rice combination were associated with a decrease in Bacteroidetes, which was mostly linked to a reduction in the genus *Bacteroides.*

In this study [76], there was no comparison to refined grain counterparts, so not much can be inferred from this study about the effects of brown rice, since it was compared to barley, which demonstrated a prebiotic effect [79]. Research needs to be done comparing brown rice to white rice and whole grain barley to refined grain barley to determine if they have differential effects on microbiota. A strength of this study was that non-targeted bacteria characterization was utilized, thus a wider array of bacteria were characterized.

### 7.4. Microbiota Studies: Mixed Whole Grains

Ampatzoglou and colleagues [77] studied the effect of increasing whole grain intake from an average of 28 g per day (~1-ounce equivalent) to an average of 168 g per day (six-ounce equivalents), compared to a refined grain diet. Despite this substantial increase in whole grain intake, which was confirmed by measuring blood alkylresorcinol concentrations, fecal microbiota did not differ significantly between the whole and refined grain diets.

In contrast to the previous study, Ross *et al*. [78] found that a combination of whole grain wheat, barley, rye and oats increased *Clostridium leptum* and tended to increase *Enterococcus* spp. compared to a diet of refined wheat, rice and maize. The highly controlled experimental diets may have eliminated confounding effects of individual background diets on microbiota.

### 7.5. Microbiota Studies: In Vitro Studies

*In vitro* studies used artificial digestion, similar to human digestion, and reaction chambers, which mimic the human intestine, where grains were reacted with human fecal slurries. While these studies should not be considered directly analogous to human studies, they do suggest that oats, rye and wheat may have an impact on human gut microbiota. Human studies are needed to test the validity of these results.

Connolly and co-workers [80] were interested in the prebiotic properties of oats and created an *in vitro* study to observe the effects of oats of different flake thicknesses with bacteria from human feces. All oats tested increased the *Bifidobacterium* genus. Thinner oats increased the levels of the *Bacteroides* and the *Prevotella* group. Short chain fatty acids increased in the incubation, and the most abundant was acetate in all conditions. With the thin oats, a significant increase in acetate and propionate was observed. Thicker oats had a significant increase in propionate and butyrate.

In a study examining rye, whole grain rye bread or boiled rye kernels were incubated with human fecal samples [81]. Following incubation, bacteria were characterized by extracting microbial DNA and amplifying it using real-time polymerase chain reactions. Both the rye bread and rye kernels increased the relative quantity of *Bifidobacterium* and decreased the quantity of *Bacteroides* compared to the original bacterial population of the inoculation feces. The rye bread reduced the *Clostridium coccoides* group.

Finally, in a study with wheat conducted by Connolly *et al.* [82], fermentation of whole wheat was carried out using an *in vitro* model utilizing anaerobic batch cultures inoculated with human feces. The wheat was either raw, partially toasted or completely toasted. Under all experimental conditions, increased numbers of the *Bifidobacterium* genus were seen. The *Lactobacillus*-*Enterococcus* group grew significantly more when given raw whole wheat flakes compared to the other experimental conditions. These findings align with the findings of the whole wheat *versus* refined wheat human feeding study [73].

### 7.6. Concluding Remarks on Whole Grains and Microbiota

Although the studies presented show that whole grains can alter the gut microbiota, there are several limitations common to the majority of studies reviewed herein. Given the relatively small sample sizes used, more studies are needed to determine how generalizable the shifts in microbiota with whole grain consumption are to larger populations. In many of the presented studies the microbiota characterization was specific to target bacteria or limited by growth medium. A broader analysis of microbiota might reveal shifts or changes in concentration in non-targeted bacteria. Furthermore, many of the experiments did not adequately assess the background diet, so confounding dietary influences may have been missed. Lastly, bacteria were often characterized at the level of phyla or family, which is too broad a characterization to make specific claims related to functionality. From the literature presented, it appears that whole grain wheat may increase the levels of *Lactobacillus*; both whole grain wheat and corn may increase the levels of *Bifidobacterium*; and whole grain barley may increase the levels of Firmicutes, particularly of the *Blautia* genus. The results from studies using a mix of whole grains were conflicting. *In vitro* studies of oats, rye and wheat showed changes in microbiota, but need to be validated using human trials. Based on the presented data, it seems likely that consumption of whole grain corn, wheat and barley does affect the microbiota; however, the mechanism behind the shift and the impact of the shift on health are still unknown. It is impossible to draw conclusions from the data at hand as to whether the observed changes lead to changes in appetite or satiety, but it remains an interesting and practical possibility.

## 8. Whole Grains and Satiety

One argument in support of the benefits of consuming more whole grains is that they are often considered more satiating than refined grains. Satiety can be defined as the absence of hunger or the sensation of being full. Consuming foods that promote satiety may have an impact on the battle against obesity [47]. In 1995, Holt and his collaborators created the “satiety index” by feeding 240 kcal servings of 38 different foods to groups of volunteers and asked them to rate their feeling of satiety every fifteen minutes for 2 h. After 2 h, the volunteers were given access to a variety of pre-weighed foods and beverages and were allowed to consume freely from the selection. The satiety index of each food was calculated by dividing the area under the satiety response curve (AUC) by that of the mean AUC for white bread. The scores were validated using the actual levels of consumption from the *ad libitum* meal. The whole grain foods tested, including whole grain wheat pasta and bread, had higher scores than their refined grain counterparts, but white and brown rice had very similar satiety scores [83].

There are a number of factors or properties ascribed to cereal grains that are believed to affect satiety, such as fiber type and concentration, acidity, buffering capacity, viscosity, bulk or physical size of the food consumed, processing techniques, such as extrusion or degradation, and macronutrient composition, to name a few [47,48,49,70,84]. As mentioned above, another factor to consider is that whole grains may contain fermentable carbohydrates that have the potential to yield SFCA in the lower bowel that can further influence satiety, either directly or indirectly. Because of the complexity of food intake regulation and the numerous known and putative factors affecting it, this topic will not be reviewed here. There are several recent reviews on this topic [85,86,87]. The studies that have been conducted on whole grains, to date, have used very simple metrics for assessing satiety. Most commonly, a self-report of sensations of hunger, fullness and other descriptors of satiety, using the visual analog scale (VAS) [87], was employed, often coupled with measurement of *ad libitum* food intake at a provided meal. In a few studies, concentrations of satiety and/or hunger signals circulating in the blood were also measured as part of the satiety evaluation [88]. The simple, comparative satiety evaluations of whole and refined grain foods is a necessary starting point for determining if indeed, whole grain foods as a group are more satiating than their refined grain counterparts [88].

Recent research studies examining widely-consumed grains are summarized in Table 2. Overall, whole oats, barley and rye were associated with increases in satiety. Whether whole wheat and maize impact satiety is not certain, because study results were mixed. No studies have been conducted in humans to evaluate the satiating properties of whole grain forms of rice.

**Table 2:** Whole grain intervention studies examining satiety.

Grain Type	Citation	Study Design	Subjects	Outcomes	Results
**Whole grain wheat compared to refined grain wheat**	Kristensen *et al*., 2010 In text citation: [89]	Randomized crossover design, 4 arms: whole grain bread refined grain bread whole grain pasta refined grain pasta Foods consumed provided 50 g of available carbohydrates and were consumed once as the test day breakfast	10 females, 6 males Age (years) 24.1 ± 3.8 BMI (kg/m^2^) 21.7 ± 2.2	- VAS assessment following test breakfast - Energy intake at *ad libitum* test lunch of single variety pizza - Glucose response following test breakfast	**Whole grain bread**: tended to increase fullness (*p* = 0.096) and satiety (*p* = 0.078) compared to the refined grain bread - No differences in *ad libitum* energy intake were seen across grain products
**Whole grain wheat compared to refined grain wheat**	Bodinham *et al.*, 2011 In text citation: [90]	Balanced randomized crossover design 2 arms: whole wheat rolls (48 g whole grain) refined wheat rolls Subjects consumed 2 test rolls for 3 weeks culminating in a test day; no rolls were consumed on the test days	9 females, 5 males Age (years) 26 ± 1.4 BMI (kg/m^2^) 21.8 ±0.8	- VAS assessment 2 fasting measures, then every 30 min post-standard breakfast for 180 min - Energy intake at *ad libitum* homogenous pasta test lunch following fiber-free standard breakfast - Week long diary of food intake (Week 3)	- No differences were observed in any tested parameter between interventions
**Whole grain oats compared to milled oat cereal**	Rebello *et al.*, 2013 In text citation: [91]	Randomized crossover design, 2 arms: oatmeal Honey Nut Cheerios™ (General Mills, Inc., Minneapolis, MN, USA), Foods consumed test cereal (250 kcal) and lactose-free, fat-free milk (113 kcal) for breakfast of the test day	29 females 17 males Age range: 18–75 years (mean: 34.1 ± 14) BMI range: 17.9–60.1 kg/m^2^ (mean: 26.1 ± 7.2)	- VAS assessment began immediately before breakfast meal, then 30, 60, 120, 180 and 240 min after the start of the breakfast meal	Oatmeal: reduced hunger (*p* = 0.0009), desire to eat (*p* = 0.0002) and prospective intake (*p* = 0.0012) and increased fullness (*p* = 0.005) compared to milled oat cereal
**Whole grain oats compared to milled oat cereal **	Rebello *et al.*, 2014 In text citation: [84]	Randomized crossover design 3 arms: instant oatmeal rolled oats (oatmeal) Honey Nut Cheerios™ Foods consumed were cereal (150 kcal) and lactose-free, fat-free milk (68 kcal) for breakfast of the test day	28 females, 20 males Age (years) 29.8 ±9.9 BMI range: 18.5- 29.9 kg/m^2^(mean 27.1 ± 6.7)	- VAS assessment measured at 30, 60, 120, 180 and 240 minutes following the test meal	Rolled oats: reduced the ratings of prospective intake more than milled oat cereal (*p* < 0.05) Instant oats: increased fullness more than rolled oats at 60 min (*p* < 0.05) increased fullness (*p* = 0.04), decreased desire to eat (*p* = 0.01), reduced prospective intake (*p* < 0.01) more than milled oat cereal over the course of four hours Rolled oats reduced prospective intake (*p* = 0.04) compared to milled oat cereal
**Whole grain barley compared to refined grain wheat**	Johansson *et al.*, 2013 In text citation: [92]	Randomized crossover design with 2 arms: whole grain barley kernels refined grain wheat bread Foods consumed once in the evening before test day; the barley kernels were given in a 96.8-g portion; the refined wheat bread was given in a 119.7-g portion	13 females, 6 males Age (years) 24.2 ±1.9 BMI (kg/m^2^) 22.3 ± 2.0	The 5.5-hour test protocol included: - VAS assessment-energy intake at *ad libitum* test breakfast (sandwich) and lunch (hash) meals - Serial breath hydrogen measures - Serial blood samples for glucose, insulin, ghrelin, GIP, GLP-1, adiponectin and free fatty acids.	Barley kernels relative to refined wheat bread: reduced perceived hunger levels following lunch (*p* < 0.05) less energy intake at *ad libitum* test lunch (*p* < 0.05) higher levels of breath hydrogen (*p* < 0.001) decreased incremental blood glucose AUC (*p* < 0.01) higher GLP-1 (*p* < 0.05) higher adiponectin (*p* < 0.05) lower serum fasting FFA (*p* < 0.05)
**Whole grain rye compared to refined grain wheat**	Isaksson *et al*., 2012 In text citation: [93]	Randomized crossover design 2 arms: whole grain rye porridge refined grain wheat bread Subjects consumed test food as a breakfast for 3 weeks Treatment breakfasts were isocaloric: Rye porridge = 193 kcal Wheat bread = 185 kcal	19 females, 5 males Age (years) 33 ± 13 BMI (kg/m^2^): 23.4 ± 2.2	The 8-hour test day included: - VAS assessment - Serial measures of breath hydrogen Subjects also completed 3 days of self- registered weighed food diaries	Rye porridge relative to refined wheat bread: subjects reported less hunger and desire to eat and higher satiety over the 4 hours after breakfast (all *p*-values ≤ 0.001); but no differences after the 4 hours higher levels of breath hydrogen between 4 and 8 hours post-test breakfast (*p* ≤ 0.05)No difference in food diaries
**Rye (various forms) compared to wheat (various forms)**	Rosen, Ostman and Bjorck, 2011 In text citation: [94]	Randomized crossover design, 7 arms: refined grain wheat bread refined grain endosperm rye bread refined grain endosperm rye bread with lactic acid whole grain rye bread whole grain rye bread with lactic acid whole grain wheat kernels whole grain rye kernels Test food were given for the breakfast meal and portioned, such that there were 50 g of available starch Foods consumed once on test day only	5 females, 5 males Age (years) 26.0 ± 1.1 BMI (kg/m^2^) 22.6 ± 0.4	The 7-hour test day included: - VAS assessment was taken every 15 or 30 min over the test day - Energy intake at *ad libitum* buffet test lunch - Breath hydrogen measured every 30 min - Serial measures of blood glucose, insulin, ghrelin - Fasting and postprandial and serum free fatty acids (FFA)	Rye kernels: Reduced desire to eat compared to all other meal types Led to less energy intake at *ad libitum* lunch compared to refined wheat bread Rye kernels and whole grain rye bread: Induced higher breath hydrogen levels in the late post-breakfast period compared to the refined wheat bread and the refined rye bread (*p* ≤ 0.05) Whole wheat kernels: reduced serum FFA compared to refined wheat bread
**Milled Whole Grain rye and whole rye kernels compared to refined grain wheat**	Ibrugger *et al*., 2014 In text citation: [81]	Randomized crossover study 3 arms: Whole grain rye bread Whole grain boiled rye kernel Refined grain wheat bread Test foods were consumed once the evening before the test day: Rye bread portion = 143 g Rye kernels portion = 147 g Refined wheat bread portion = 111 g	12 males Age (years) 25.6 ± 3.9 BMI (kg/m^2^) 23.1 ± 1.2	Measurements included: - VAS satiety assessment before and after the evening test meal and during the following test day - Breath hydrogen was measured over the course of the test day - Energy intake at *ad libitum* pasta Bolognese test lunch	No differences in VAS satiety between grain treatments Whole grain rye bread and rye kernels: increased breath hydrogen (*p* < 0.01 and *p* < 0.05, respectively) Reduced energy intake at lunch (by 11% *p* < 0.01 and by 7% *p* < 0.05, respectively) compared to refined wheat bread
**Whole grain corn at low and high levels compared to refined grain wheat**	Luhovyy *et al.*, 2014 In text citation: [95]	Randomized crossover design, 3 arms: High amylose maize flour was mixed with refined wheat flour control: 100% refined wheat low-dose: 63% refined wheat + 37% maize high-dose: 33% refined wheat + 67% maize Test food consumed as a cookie between breakfast and lunch once on the test day only	30 males Age (years) 22.9 ± 0.6 BMI (kg/m^2^) 22.6 ± 0.3	- VAS assessment before and after consumption of test cookie - Energy intake at *ad libitum* test lunch featuring a variety of pizzas	No effects of maize flour at either level on VAS satiety assessment or energy intake at *ad libitum* test lunch between interventions
**Whole grains wheat whole grain barley refined grain rice**	Schroeder *et al.*, 2009 In text citation: [96]	Randomized crossover study, single blind, 3 arms: whole wheat hot cereal whole barley hot cereal refined rice hot cereal Test foods were consumed once on the test day only: Wheat cereal portion = 220 kcalBarley cereal portion = 200 kcal Rice cereal portion = 210 kcal	35 females, 12 males Age Range: 19–58 years (mean: 31 ± 11)B MI Range: 18.8–30.7 kg/m^2^ (mean: 23 ± 3)	- VAS combined with the Satiety Labeled Intensity Magnitude Scale (SLIM) was measured at Time 0 and at 120, 130, 210 and 240 min after breakfast - Energy intake at *ad libitum* smorgasbord test lunch	No difference in self-reported satiety scores between treatments Barley cereal: Overall VAS/SLIM scores for hunger were lower before lunch compared to before breakfast (*p* = 0.002)No differences between treatments in energy intake at *ad libitum* lunch

### 8.1. Satiety Studies: Comparison of Whole versus Refined Wheat

Studies examining the effect of whole grain and refined grain wheat on satiety are limited to one short-term study with grain products eaten on a single occasion [89] and one intervention study with grain products consumed for three weeks prior to assessment [90]. In the short-term study, the wheat products included bread or pasta and each subject ate four meals on separate occasions: whole grain bread, refined grain bread, whole grain pasta and refined grain pasta. When in the bread matrix, whole wheat bread significantly increased fullness and satiety compared to the refined grain bread, but no corresponding decrease in *ad libitum* energy intake at a provided lunch meal was recorded. In the intervention study, whole grain wheat rolls, providing 48 g of whole grain per day, were consumed daily for three weeks, and this intervention was compared to consuming refined grain wheat rolls daily. At the end of the intervention periods, no differences in self-reported satiety or energy intake at the *ad libitum* lunch meal or overall dietary intake were found between the whole wheat and refined wheat condition. Accordingly, body weight, percent body fat, waist or hip circumference did not differ in response to the whole or refined wheat intervention.

### 8.2. Satiety Studies: Comparison of Oats with Different Levels of Processing

Two studies were performed using oatmeal and oat flour-based ready-to-eat breakfast cereal. Both found that oatmeal was significantly more satiating than the more highly processed oat flour breakfast cereal.

Rebello and co-workers [91] conducted research on the satiating effects of Quaker Old Fashioned Oatmeal (Quaker Oatmeal from Pepsico Inc., Barrington, IL, USA), compared to the most widely-sold ready-to-eat breakfast cereal (RTEC), Honey Nut Cheerios™. The RTEC is made with whole grain oat flour, while the Old Fashioned Oatmeal is made from rolled oats, so while there is no refined grain product tested in this trial, the RTEC does present a more highly processed version of the grain than the oatmeal. When consumed along with fat-free, lactose-free milk, oatmeal was associated with a reduction in hunger, desire to eat and prospective intake and an increase in fullness compared to the RTEC. The researchers attribute these findings to the higher fiber and protein content and lower sugar content in the oatmeal compared to the RTEC.

A second study by Rebello *et al.* [84] included an assessment of instant oatmeal (Quaker Instant Oatmeal Flakes™, Quaker Oatmeal from Pepsico Inc.), along with the Old Fashioned Oatmeal™ (Quaker Oatmeal from Pepsico Inc.) and Honey Nut Cheerios™ (RTEC). Instant oatmeal increased fullness and suppressed the desire to eat and ratings of prospective intake significantly more than the RTEC. The Old Fashioned Oatmeal reduced the ratings of prospective intake significantly more than the RTEC. Consumption of instant oatmeal increased fullness more than Old Fashioned Oatmeal at 60 min, but there was no significant difference seen in satiety measures between the oatmeals over the full four-hour study period. In this study, the satiety effects associated with oatmeal were similar, but less marked, than those reported above [91]. Possible explanations for the difference between the two studies are that the difference in portion size (the two-arm study provided 66% more calories than the three-arm study) had an effect on satiety. Honey Nut Cheerios™ may not be an ideal control because of the sugar and protein differences between the RTEC and oatmeals. As part of these studies, chemical assays to determine compositional differences of the cereals were done, and the cereals were subjected to oral and gastric *in vitro* digestion procedures [91]. Both forms of oatmeal had a higher molecular weight, greater viscosity and larger hydration spheres, as well as having a higher β-glucan content. Instant oatmeal and the Old Fashioned Oatmeal were not significantly different from each other, but the instant oatmeal had greater oral and initial gastric viscosity compared to the RTEC. Since instant oatmeal is more thinly cut, it may be more easily rehydrated, which would mean that its viscosity would increase more quickly, as it is able to absorb water faster [84]. As the *in vitro* digestion continued, the Old Fashioned Oatmeal viscosity was higher, compared to the RTEC, corresponding to the time (120 min) when differences in satiety were first observed in the human trial [91]. This difference in viscosity was likely due to either the higher fiber level or, more specifically, the greater concentration of β-glucan, the main component of soluble oat fiber in oatmeal [97]. The larger hydration spheres would lead one to believe that the soluble fiber in the oatmeal was able to hydrate more quickly than the soluble fiber in the RTEC, which may also be a factor leading to the higher viscosity in the oatmeal [91]. Viscosity is important to satiety, because it affects the transit time of food in the digestive system and leads to longer lasting feelings of fullness and less feelings of hunger, both of which are mediated via hormonal controls [47].

### 8.3. Satiety Studies: Comparison of Various Rye Products with Reference to Refined Wheat

While there are many studies evaluating the satiating effects of rye, three recent studies utilizing healthy adults are presented here. In one study, a breakfast of whole grain rye porridge increased satiety for four hours after consumption compared a breakfast of refined grain white bread. In another study, a whole grain rye kernel breakfast increased satiety and led to decreased energy intake at a lunch meal. Finally, the results of a third study demonstrated that an evening meal of whole grain rye increased morning breath hydrogen production and decreased energy intake at a lunch meal. In the case of rye, the level of refining plays a role in satiating effect, with less refined products, such as rye kernels or porridges, having a stronger satiating effect than milled rye flours.

Isaksson and co-workers [93] evaluated the satiety effect of a whole grain rye porridge or a refined grain wheat bread breakfast. The two different grain treatments were eaten daily at a breakfast meal for three weeks. Less hunger and desire to eat and greater satiety were reported up to four hours after consuming the rye porridge at breakfast. Furthermore, the rye porridge intervention produced higher levels of breath hydrogen, but breath hydrogen did not correlate with reported hunger or satiety. The increase in satiety following rye porridge ingestion could have resulted from the high level of fiber, the increased water load associated with the porridge and, possibly, the increased stomach distension due to the larger volume of the porridge compared to the bread. In a previous study, the satiating effect of whole rye was seen over eight hours, as opposed to the four hours seen here; this could be caused by differences in portion size and preparation of the rye [98,99].

A randomized multi-crossover study was conducted to test the effects of seven different rye or wheat products consumed as a breakfast meal: refined grain wheat bread, refined grain endosperm rye bread, refined grain endosperm rye bread with lactic acid, whole grain rye bread, whole grain rye bread with lactic acid, whole grain wheat kernels and whole grain rye kernels [94]. The rye products beneficially increased reported satiety in the early and late postprandial periods. Rye kernels increased satiety most dramatically, both acutely and in the face of a second meal, as demonstrated by the lower energy intake at lunch and the self-reported VAS scores compared to the breakfast of refined wheat bread. Furthermore, there was an overall decrease in calories consumed on the rye kernel test day compared to the refined wheat bread test day [94]. As noted above for the Isaksson study, the difference in the amount and volume of these different grain products needed to achieve isocaloric portions could play a role in the satiating effects when comparing cereals to breads [93,94]. The ability of fibers in the kernels to hydrate and hold water from the cooking liquid could create increase bulk in the cereals, thereby changing gastric distension and hormonal or mechanoreceptor-mediated signals of satiety [47]. However, this mechanism would not explain the second meal satiety effect seen with the rye kernel breakfast. The investigators posited that increased microbial fermentation led to an increase in fermentation metabolites, which was suggested by increased breath hydrogen measured after the whole grain rye bread as compared to the refined grain wheat bread conditions. These fermentation products may improve glucose regulation and increase satiety, possibly by delaying the release of the hunger hormone, ghrelin [94].

Whole grain rye bread and boiled rye kernels were compared to a refined wheat bread in a study conducted by Ibrugger and colleagues [81]. The grains were fed as part of the evening meal. Self-reports of satiety, *ad libitum* intake at a lunch meal and breath hydrogen measurements were made the following day. Although no differences in self-reported satiety were found between treatments, energy intake at the *ad libitum* lunch did differ with both rye bread and rye kernels leading, to a reduction of energy intake compared to the refined wheat bread. Breath hydrogen levels, an indicator of fermentation, were significantly higher with the rye treatments, as well [81]. Although the satiety self‑reports did not yield differences between treatments in this study, which is in contrast to the satiety reported in the Isaksson study, differences in study design, feeding time and test foods may account for these differences [93]. While the Ibrugger study was not able to draw a conclusion that included a clear causal factor linking satiety and microbiota, due in no small part to the separation of the satiety trial, conducted in humans, and the microbial fermentation, conducted *in vitro*, it does suggest that an increase in *Bifidobacterium* and a decrease in *Bacteroidetes* may be tied to the decrease in energy intake seen with whole grain rye consumption [81].

### 8.4. Satiety Studies: Comparison of Whole Maize *versus* Refined Wheat

Luhovyy and coworkers [95] evaluated cookies prepared with whole grain high amylase maize flour (HiMaize^®^, Ingredion Incorporated, Bridgewater, NJ, USA) at two levels: a higher dose of 53.5 g, a lower dose of 43.5 g, compared to cookies containing refined wheat flour only. The satiety assessment included self-reported satiety and *ad libitum* food intake following ingestion of the experimental cookies, which were consumed after a standard breakfast. The maize treatments had no effect on the measures of satiety under the conditions of this study. A milled oat cereal (Honey Nut Cheerios™) was included in the standard breakfast, possibly confounding any satiating effect of the maize.

### 8.5. Satiety Studies: Comparison of Barley Kernels to a Reference Condition of Refined Wheat

Johansson and co-workers [92] observed the effects of barley on food intake, appetite and glucose metabolism over the course of 16 hours. Subjects consumed either whole grain barley kernels or refined grain white wheat bread in the evening and then they fasted overnight. The test protocol began the following morning and included observing *ad libitum* food intake at breakfast and lunch meals, collecting self-reports of satiety and monitoring blood glucose and endocrine responses in response to the meals. Evening consumption of the barley kernels resulted in decreased hunger the following day and less energy intake at the provided lunch meal. Consistent with the reported decrease hunger, circulating GLP-1 concentrations were higher with the barley ingestion. The barley kernel consumption resulted in higher breath hydrogen levels compared to the refined wheat bread, suggesting that microbial fermentation of the indigestible carbohydrate components of barley may have contributed to the satiety response.

### 8.6. Satiety Studies, Comparison of Cereals: Whole Grain Wheat, Whole Grain Barley and Refined Rice

In a study that compared satiety responses to cooked cereals consisting of whole grain wheat, whole grain rye or refined rice consumed at a breakfast meal, no differences were found in overall self-reported satiety scores or *ad libitum* intake of energy or macronutrient intake at a lunch meal [96]. Consumption of the barley cereal decreased hunger reported before lunch as compared to hunger reported before breakfast, but the other cereals did not have a similar effect.

## 9. Conclusions and Considerations for Future Studies

Dietary staples, such as rice and corn, have been overlooked as research targets for satiety studies. The satiety literature would benefit from studies covering a full array of commonly-consumed grains. Rye, a staple of Northern Europe, has an established body of literature associated with its effect on satiety. Gaps, such as the effect on microbiota, exist, but overall, the level and depth of research on rye is a good model for what needs to be done to test for satiety effects of other grains.

There is a dearth of information on general health effects of combinations of whole grains, even though in practical terms, most human diets are likely to include more than one type of grain. Synergistic effects of whole grains have not been studied systematically, and thus, the phenomenon, if it exists, is poorly understood. When a combination of different grains is consumed, this will expand the types of fiber, micronutrients, polyphenols and other bioactives entering into the gastrointestinal tract. Theoretically, these components may act synergistically and yield greater bacterial diversity or induce a wider array of metabolic pathways in resident bacteria [100]. The idea that combinations of whole grains might have a more profound impact than single grains was touched upon in the Martinez paper presented. While they did not see improvements in satiety, or a change in body weight, they did observe a stronger effect of the combination of whole barley and brown rice on decreasing pro‑inflammatory markers than either grain individually [76]. Inflammation is integrally linked to obesity, so perhaps a longer intervention may have yielded some effects on body weight [101].

Milling, or the level of processing, may be an important factor to consider when interpreting results of whole grain studies. Milling can affect the activity of bioactives, as well as the availability of carbohydrates and fibers [102]. Considering the presented studies on rye, there were stronger satiating effects of whole rye kernels than whole rye flour, even thought they should have a similar composition. Studies are needed to evaluate the effect of milling of other grains on satiety. Milling affects the size of the grain particle that reaches the intestine, and particle size might dictate its use as a substrate, thereby potentially enriching, shifting or altering the metabolism of the microbiota. Americans consume very little whole grain foods, and the little they do consume is primarily in the form of breads, pastas, cooked cereal and ready-to-eat breakfast cereals [21]. These foods represent a wide range of milling, from the highly processed flours needed to make yeast breads to very minimally processed cooked cereals. If milling proves to reduce the health benefits of whole grains substantially, this message should be disseminated to consumers to provide a strong health incentive for preferentially increasing the consumption of minimally-processed grains.

Understanding and parsing the link between microbiota, their fermentation products and satiety is still in its infancy. The mechanisms proposed in this paper are by no means definitive and require more research to demonstrate a reliable connection. The results of satiety studies in humans are often inconsistent as to why a food was satiating in one trial and not in another [93]. Satiety is a complex, multifaceted and highly subjective sensation, and results will depend on how it is assessed, the context in which it is assessed and the personal attitudes, beliefs and behaviors surrounding foods held by individuals providing the assessment. To study satiety, and how microbiota may influence it, study designs must use the appropriate control and employ a cross-over design to account for fixed effects associated with individual participants. Furthermore, important elements of the studies would be to characterize microbiota under well-defined dietary conditions and to determine if fermentation products, like SCFAs, or other products of microbial metabolism play a role in satiety.

Once our understanding of the impact of whole grains on satiety and microbiota is more developed, then particular grains, their fiber components or combinations of grains might be applied to assist with better appetite control. With the advent of better, faster and less expensive sequencing technology, microbial characterization has never been more within reach at an individual level. In the future, with this information at hand, healthcare professionals could make individual dietary recommendations that promote satiety and contribute to weight control.
